# Monitoring compliance of CITES lion bone exports from South Africa

**DOI:** 10.1371/journal.pone.0249306

**Published:** 2021-04-02

**Authors:** Vivienne L. Williams, Peter G. Coals, Marli de Bruyn, Vincent N. Naude, Desiré L. Dalton, Antoinette Kotzé

**Affiliations:** 1 School of Animal, Plant and Environmental Sciences, University of the Witwatersrand, Johannesburg, South Africa; 2 Department of Zoology, Wildlife Conservation Research Unit, Recanati-Kaplan Centre, University of Oxford, Oxford, United Kingdom; 3 South African National Biodiversity Institute, Pretoria, South Africa; 4 Institute for Communities and Wildlife in Africa, University of Cape Town, Cape Town, South Africa; 5 Department of Genetics, University of the Free State, Bloemfontein, South Africa; Pingtung University of Science and Technology, TAIWAN

## Abstract

From 2008 to 2018, South Africa permitted the export of captive-bred African lion (*Panthera leo*) skeletons to Southeast Asia under CITES Appendix II. Legal exports rose from approximately 50 individuals in 2008 to a maximum of 1,771 skeletons in 2016, and has led to ongoing concerns over possible laundering of non-lion, multiple-source and wild-sourced bones. South Africa is required under its obligations to CITES to employ mechanisms for monitoring and reporting trade, and to limit the potential for illegal trade and laundering of lion and other large felid bones. Monitoring tools for legal trade are critical to compliance with CITES. Here we evaluate the CITES-compliance procedure implemented by South Africa for export of lion bones and identify six essential general points for consideration in the implementation of animal export quota compliance protocols. We provide specific insight into the South African lion bone export monitoring system through: i) outlining the protocols followed; ii) assessing the utility of cranial morphology to identify species; iii) evaluating skeleton consignment weight as a monitoring tool; and iv) presenting molecular (DNA) species assignment and pairwise-comparative sample matching of individuals. We describe irregularities and illicit behaviour detected in the 2017 and 2018 lion bone quotas. Notably, we report that the compliance procedure successfully identified and prevented the attempted laundering of a tiger (*P*. *tigris*) skeleton in 2018. We emphasise the utility of mixed-method protocols for the monitoring of compliance in CITES Appendix II export quota systems.

## Introduction

Legal and managed trade of wildlife resources is highly contested. Detractors argue that it is unethical and detrimental to threatened species while, conversely, advocates maintain that trade bans are ineffective and have, in the case of some species, increased incentives for illegal exploitation thereby failing to stem the decline of targeted wild populations [1]. The legal sale of African lion (*Panthera leo*) bones and body parts is at the centre of one such debate [2–5] and encompasses not only conservation concerns, but also animal welfare, stakeholder livelihoods, and international politics [5,6].

From 2008 to 2018, South Africa permitted the export of lion skeletons to Southeast-Asia [2–4]; these skeletons originated from captive-bred lions that were either hunted or euthanized [4]. International wildlife trade is governed by the Convention on International Trade in Endangered Species of Wild Fauna and Flora (CITES) which lists lions on Appendix II, permitting trade at a level that is not detrimental to the survival of wild lion populations [7,8]. Numbers of legally exported skeletons originating from South Africa rose from approximately 50 individuals in 2008 to a maximum of 1,771 skeletons in 2016 [3,4,9].

Concerns around the legal trade in lion bones include, amongst other issues, the laundering of wild-sourced skeletons [5]. At the CITES Conference of the Parties in 2016 (CoP17) consensus on a proposal by nine African Parties to transfer lion from Appendix II to Appendix I could not be reached [4,10,11]. Instead, through discussions in a working group, a compromise was reached—i) lions remained on Appendix II (i.e., exports of lion parts are allowed for commercial purposes), but with the annotation that, as a Party to CITES, South Africa is obligated to establish ii) an annual export quota for trade in bones, bone pieces, bone products, claws, skeletons, skulls and teeth, derived from captive breeding operations, and iii) a zero annual export quota for such derivatives removed from the wild and traded for commercial purposes [6].

Guided by the scientific advice of the South African CITES Scientific Authority, coordinated by the South African Biodiversity Institute (SANBI), the South African government, through the Department of Environment, Forestry and Fisheries (DEFF) set the first export quota at 800 lion skeletons in 2017 [4], and maintained it at 800 in 2018 [5]. No skeletons were legally exported in 2019 due to legal action taken against DEFF by the NSPCA (RSA: Case #86515, 2019). No quota was set for the export of partial derivatives (e.g., bone pieces, teeth, claws or individual bones). CITES requires that: “*every Party that has established an export quota is responsible for monitoring its use and must ensure that it is not exceeded*. *For that purpose*, *it should maintain data on the number or quantity of specimens actually exported*, *to be used as a reference when reviewing applications to authorize further exports*” [12]. Thus to be compliant with CITES, South Africa was required to employ mechanisms for monitoring and reporting trade. Such procedures aim to limit the potential for illegal trade and laundering of lion bones (and those of other large felids), by ensuring that legal exports of captive-bred lion skeletons through the quota system are not used as conduits to export i) more skeletons than permitted, and ii) the bones and derivatives of wild lions, tigers (*Panthera tigris*; international commercial trade prohibited), and hybrids such as ligers (*P*. *leo* x *tigris*).

Cranial and mandibular morphology offers the first practical level of species differentiation among lions and tigers [9], provided skeletons are complete, thereby equipping compliance officers with a provisional specimen identification tool during random spot-checks. However, in the absence of morphological features and the capacity to distinguish between species or possible hybrids, DNA analysis with a forensic approach is an essential investigative tool used in wildlife law enforcement to provide conclusive evidence for species identification [13].

DNA profiling techniques include, amongst others, the application of nuclear and mitochondrial DNA markers, developed for revealing inter- and intra-species genetic variation, providing information on the species and individual being traded. DNA barcoding using mitochondrial DNA (mtDNA) loci such as cytochrome c oxidase I (COI) and the control region (CR), as well as 12S and 16S ribosomal RNA (rRNA) have been successfully used for species identification [14–17]. In addition, nuclear markers can reliably be used to differentiate between individuals [18–22]. The conserved marker flanking regions across taxa, such as those developed for the domestic cat (*Felis catus*), allows for amplification across related species, such as lion and tiger [19,23]. These techniques, when verified, can be harnessed for forensic applications with the potential to detect skeletons of non-lion species and multiple individuals being laundered through the legal trade chain.

Lion skeleton weights provide an additional tool for monitoring compliance. However, in light of substantial additions of individual known-weight skeletons to the monitoring data set of Williams et al. (2015) [9] over five years, and observed changes in the broader lion trade industry, international politics and consumer demand, the utility of weight as a predictive compliance tool requires re-evaluation.

To our knowledge, no peer-reviewed publications have evaluated case-specific processes of CITES Appendix II export quota implementation and compliance for captive-bred species. To address this information paucity, we reviewed the DEFF-implemented compliance process for the legal export of lion skeletons from South Africa in 2017 and 2018 under the mandated quota, and highlighted generalities that may apply to other traded species and contexts. By describing the export quota system implemented by the DEFF, and the results of trade monitoring, the aim of this study is not to justify the lion bone trade–instead, we provide insight into the adopted mixed-methods monitoring system through: i) outlining the DEFF-instituted principles, protocols and steps; ii) assessing the utility of cranial morphology to identify species; iii) evaluating skeleton weight as a rapid consignment monitoring tool; iv) presenting molecular species assignment and pairwise-comparative sample matching of individuals; and v) describing irregularities and detecting illicit behaviour therein.

## Materials and methods

### Ethical clearance

**Fig 1:**
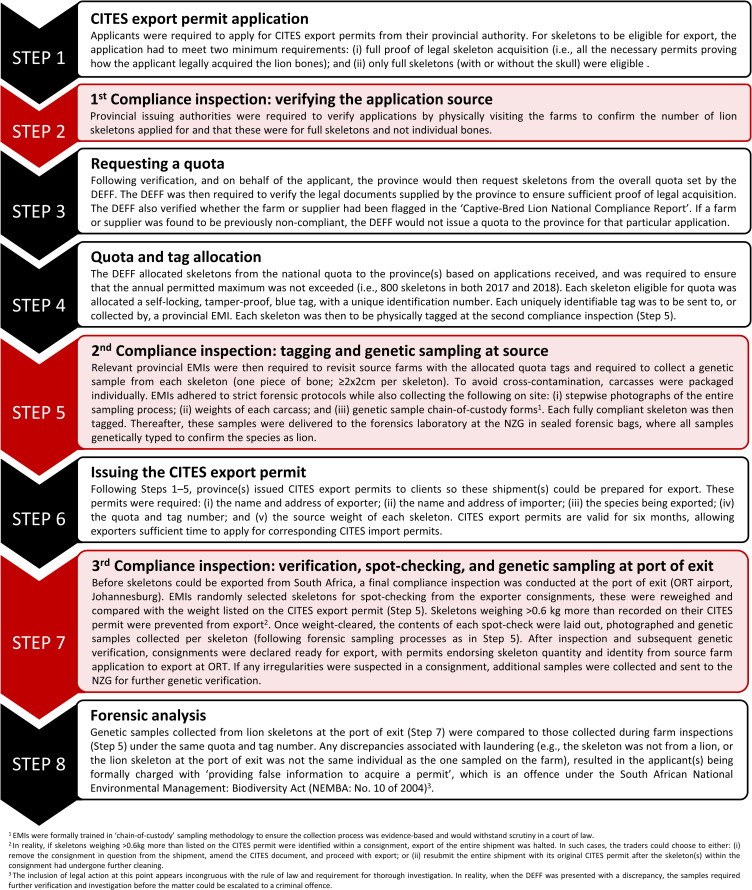
Eight-step compliance procedure devised by DEFF for implementing the legal lion bone export quota from South Africa in 2017 and 2018 (Source: DEFF, pers. comm., 2019). Sequential steps from permit application submission by source farms and traders to physical export from O.R. Tambo (ORT) airport are provided, indicating the three mandatory compliance inspections (red).

Information was provided or mined from government databases, repositories and sources. Hence, animal ethics clearance was not required as no lions were specifically killed for this study. The DEFF required all lion skeletons eligible for export to Southeast Asia to be DNA tested and weighed at least once (Fig 1, Steps 5 and 7).

### Source farm sampling and airport spot-checks

The DEFF employed the following six principles intending to secure the export process from laundering within the legal trade chain (DEFF pers. comm. 2019): i) only skeletons of captive-bred lions may be exported under the quota (therefore exports of tigers and lion-tiger hybrids are prohibited); ii) there is a zero export quota for lion parts sourced from wild lions; iii) only full skeletons (with or without skulls) can be exported; iv) the quota is controlled by the DEFF (as the CITES Management Authority) and is issued to applicants on a first-come-first-serve basis; v) bone samples from all the skeletons are to be taken at the source (farm/facility) for DNA species confirmation (the exporter pays ZAR 660 [U$ 40] per test), and bone samples taken from spot-checked skeletons at the port of exit for DNA matching (paid by the DEFF); and vi) all skeletons that qualify for the quota must be tagged by a provincial EMI (Environmental Management Inspector). Governed by these principles, an eight-step compliance procedure (Fig 1) was developed by the DEFF for implementing the lion bone quota.

**Fig 2:**
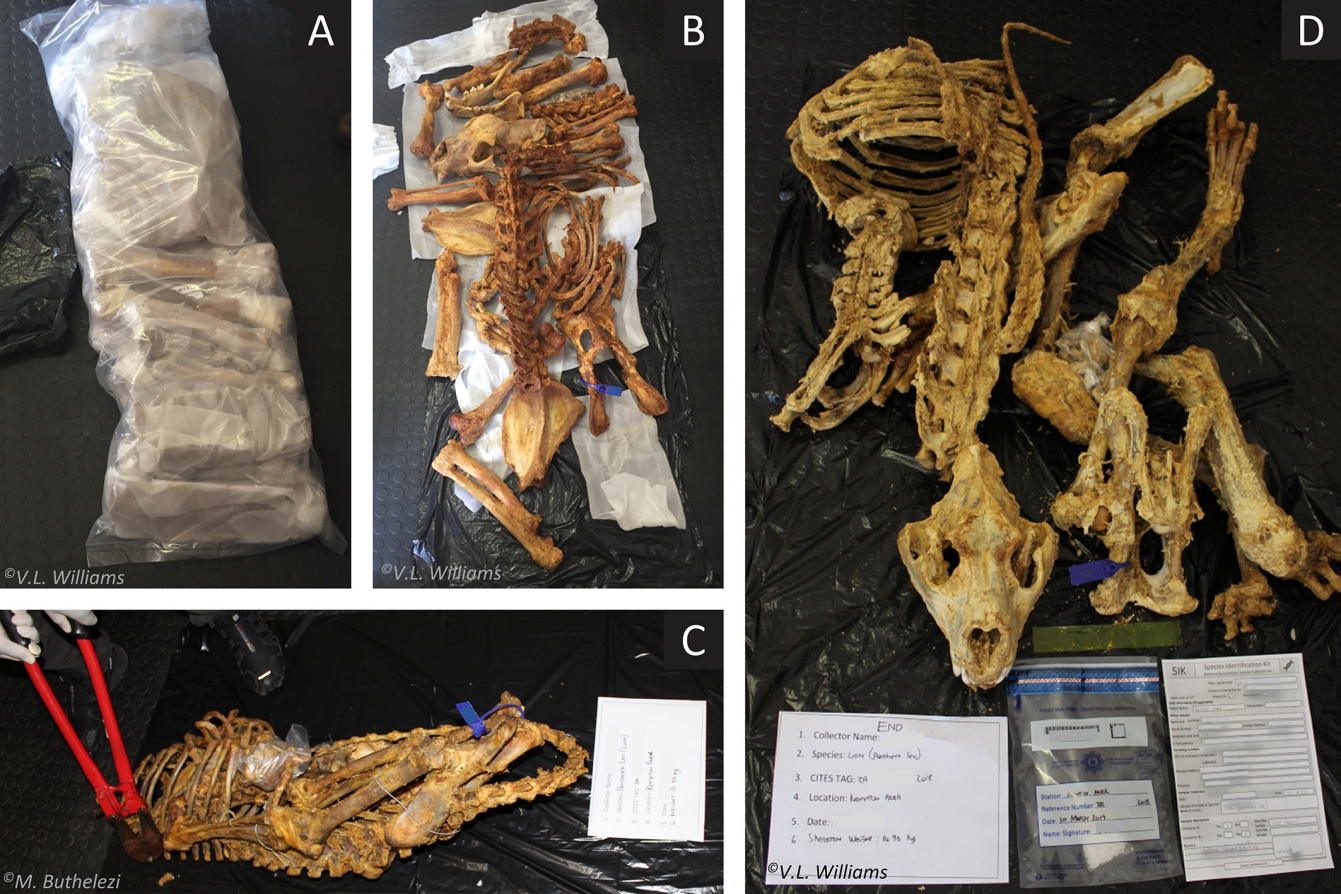
Spot-checked skeletons (12.5% of every consignment) during a 3^rd^ compliance inspection at ORT airport in 2018 (Step 7). All checked bags were reweighed, inspected, photographed and had a sample taken for DNA species assignment and pairwise-comparative sample matching. The lion skeleton in (A) and (B) was the heaviest (38.0 kg) of all the skeletons weighed at their source farm (Step 5) in 2018, but only the third heaviest (20.5 kg) on export (Step 7). (C) Collecting a sample following the forensic protocol for genetic testing (farm = 14.0 kg; spot-check = 10.35 kg). (D) A completed inspection (19.2 kg; 14.9 kg).

The DEFF made improvements to the permitting, inspection, compliance, verification, and sampling to ensure more optimal processes. In 2017, for example, there was no formalized sampling strategy to decide how many skeletons to spot-check at the point of exit; hence 25 skeletons were randomly selected during the period of consignment export (Fig 2). In 2018, however, the SANBI laboratory and an EMI/DEFF official at the airport revised the airport sampling strategy by implementing random spot-check sampling that amounted to at least one-eighth (12.5%) of the 800 skeletons in the quota being included in the verification testing as well as at least one per batch. Accordingly, 12.5% of every exporter’s consignment was randomly spot-checked and sampled at the point of exit. In 2018, 102 samples were taken at the airport; an additional sample was collected at the airport from one skeleton for verification as prior anomalies had been identified in one farm specimen with the same tag number. Also, in 2018, morphological characteristics on the crania and mandibles of randomly selected skulls were visually examined to assess whether they were lion or tiger.

### Skull morphology

**Fig 3:**
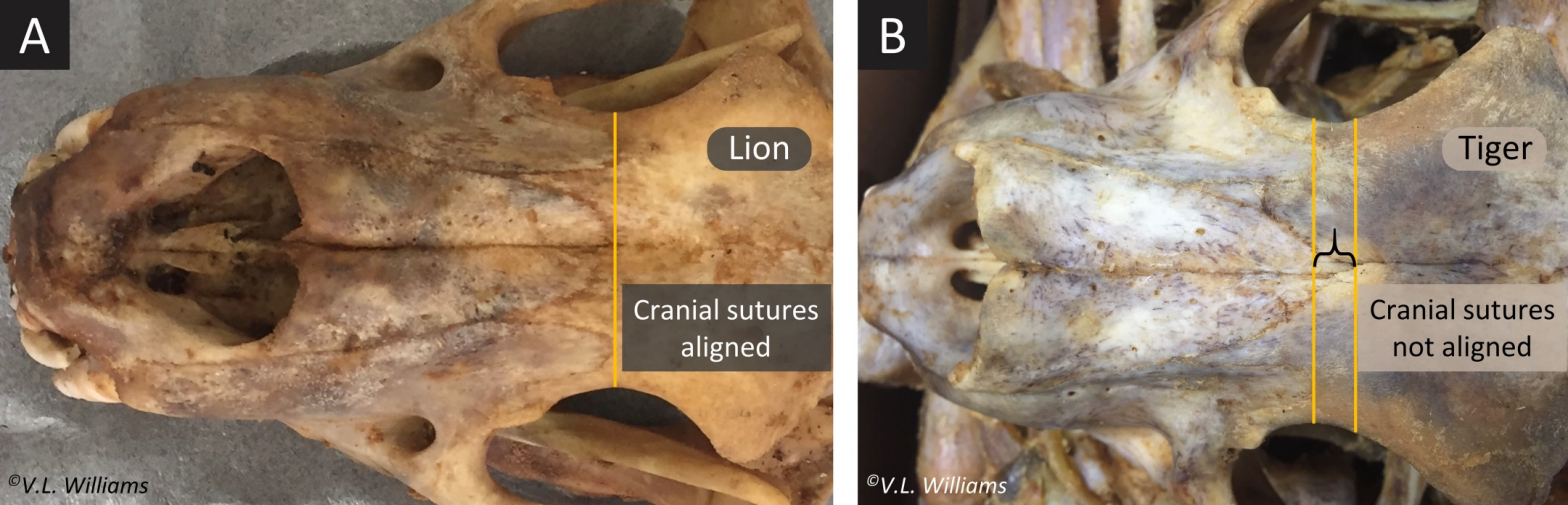
Spot-checked skulls were of (A) lions (skeleton in Fig 2A and 2B), except for one specimen in 2018, which resembled that of a (B) tiger (skeleton in Fig 4E). These are distinguished using the alignment of the posterior projections of the nasal-frontal and maxilla-frontal sutures: the sutures align in lions but not tigers. Also, unlike lions, tiger mandibles do not rock when placed on a flat surface [9].

There are diagnostic differences in the skull morphologies of lions and tigers that are illustrated by the mnemonic “*lions align and their mandibles rock*” [9]. In lions, the posterior projections of the nasal-frontal sutures on the cranium are aligned with the apices of the maxilla-frontal sutures (Fig 3A), and the ventral profiles of the mandibles are convex causing a rocking motion when placed on a flat surface. In tigers, however, the cranial sutures do not align (Fig 3B), and the mandibles are concave or straight and do not rock on a flat surface. Infrequent exceptions to this diagnostic difference for lion mandibles aside (V.L. Williams, pers. obs., 2019), using this rapid and low-cost methodology has been proposed as a provisional tool for confirming species in compliance inspections. EMIs were trained in 2018 to recognize these morphological differences and provisional species identifications using comparative skull morphology were made for skeletons.

### Skeleton weight

To determine the efficacy of using lion skeleton weight as a monitoring tool and evaluate claims by Pickover (2019) [24] of attempted laundering, consistent and predictable weight profiles were required across the quota by location (source farm and airport, hereafter ‘farm’ and ‘port’ respectively). Weight profiles were therefore examined to (i) address laundering claims, and (ii) indicate significant changes that may confound the use of weight as a monitoring tool.

**Fig 4:**
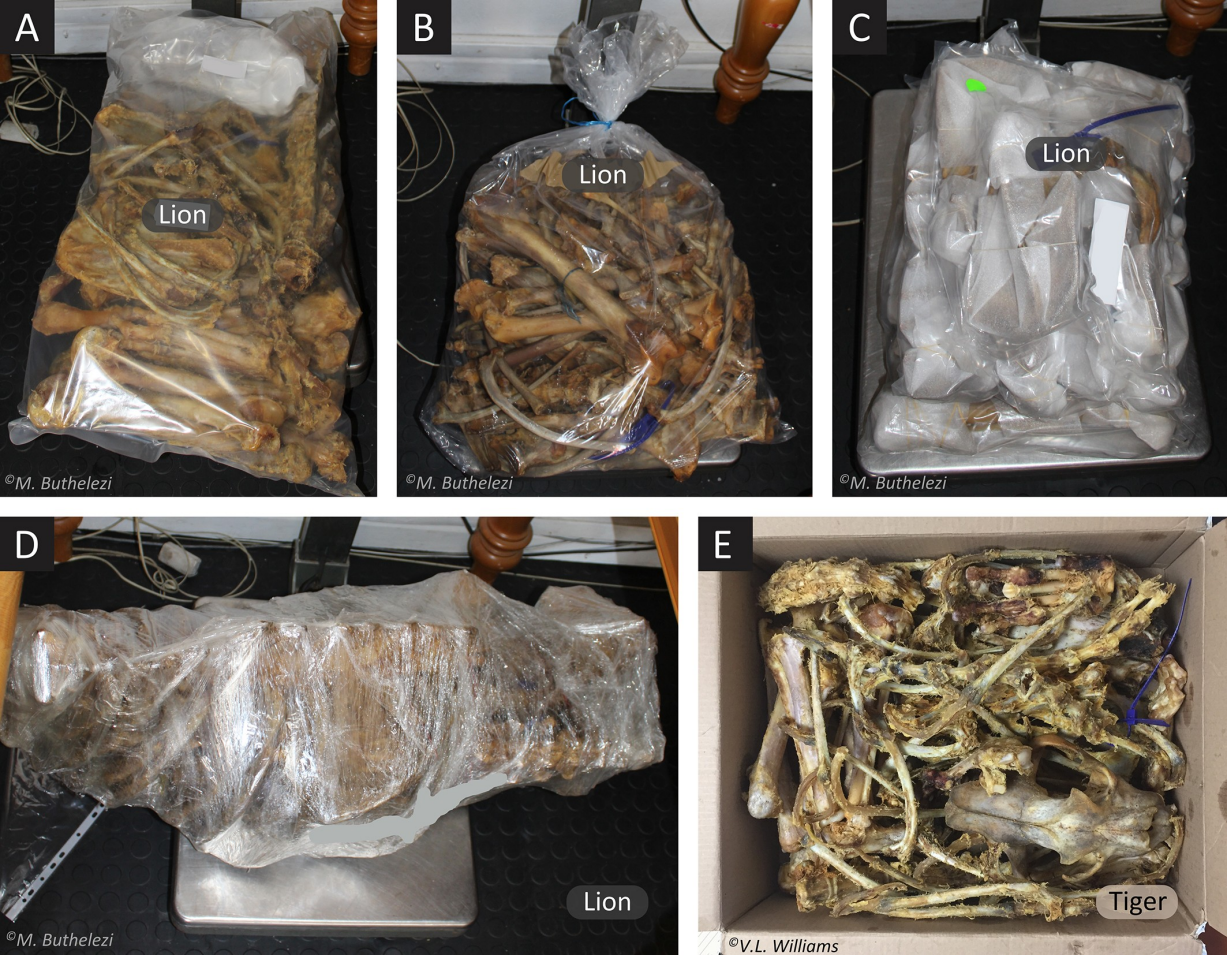
(A–D) Export-ready lion skeletons, processed and packaged in various ways, as reweighed at O.R. Tambo (ORT) airport during the 3^rd^ compliance inspection (Step 7). (E) A suspected tiger skeleton confiscated at the port. Following DNA-based species assignment of *P*. *tigris* on a skeleton with the same tag number at the source farm (Step 5), this skeleton was confirmed as a tiger (by tag and skull morphology).

Skeleton weight was recorded for 1,600 skeletons (*n*_2017_ = 800; *n*_2018_ = 800) on farms by compliance inspectors (EMIs). Spot-checked individuals (*n*_2017_ = 25; *n*_2018_ = 102 randomly selected at ORT airport by EMIs, were reweighed (including various packaging) and compared to their corresponding farm weight (Step 7). Spot-checked consignments were also opened by EMIs, physically checked for multiple individuals (i.e., bones of >1 lion), and a (rib bone) sample taken for genetic analysis. These procedures were also systematically photographed (Fig 4). Where differences in corresponding weights of skeletons were found (port weight >0.6 kg heavier than farm weight), further investigation was conducted.

While all documentation required for compliance was available upon inspection (Steps 2, 5 and 7), some farm (*n*_2017_ = 33, *n*_2018_ = 15) and port weight (*n*_2017_ = 25) data were not retained by EMIs post-inspection, as this was not required in 2017. While photographs (showing consignment weights) were taken at the 2017 port spot-check, these tag numbers were not completely visible, precluding any pairwise comparative analyses; however, an exporter provided data on request for their consolidated exports in 2017 (i.e., 123 skeletons from six consignments), including (i) endorsed addendums to the CITES export permits, detailing the farm weight and allocated tag numbers per skeleton, (ii) air waybills (AWBs), indicating the total weight of each consignment, and (iii) the packaging weight per consignment, allowing for the calculation of nett consignment weights. Albeit biased to a single exporter, these data are comparable to the port spot-check sampling of 2018. Two further temporal comparisons of these annual consignment weights (AWB information on exports to southeast Asia; 2014–2016) were sourced, under exporter consent, from the freight forwarding company which handles most consignments [3]. Hence, all farm weight analyses were conducted with 1,551 lion skeletons (instead of 1599), and pairwise-comparisons between farm and port weights of spot-checked specimens with 102 skeletons inspected in 2018, using only DEFF data (see S1 Table for sample number summary). All available photographs taken during inspections (2017–2018) were further scrutinized, especially where irregular skeleton weights were reported.

We investigated whether skeleton weight differed significantly between farm and port, and if mean skeleton weights differed significantly across years (i.e., does the regression presented in Williams et al. (2015) [2] for weight up to 2014 hold for skeleton weight estimations in the period after 2014). The i) mean farm and port skeleton weights were compared using a paired t-test, ii) difference in mean skeleton weight between traders was tested for using an ANOVA, iii) number of skeletons were plotted against consignment weights and linear regressions fitted for the years (a) up to 2014, (b) 2016 and (c) 2017 and 2018 combined, and iv) difference between mean skeleton weight between years was tested for using an ANOVA. All statistical analyses were conducted in *R* [25].

### Molecular analyses

Although not strictly a method of this paper, we detail the molecular analyses conducted by the SANBI laboratory as part of the CITES compliance protocol. A complete and transparent understanding of the forensic methodology pursued by the laboratory is necessary to effectively evaluate the process of compliance that follows in the Results and Discussion.

Molecular species verification and pairwise-comparative sample matching were conducted on lion bone samples to confirm that: i) the species is lion, and ii) farm and port samples match (i.e., sample pairs collected at farms match those spot-checked at ports and originate from the same individual).

Bone samples were collected using a s*pecies identification kit* (i.e., forensic evidence bags, unique tags, preservatives and datasheet) provided by the DEFF, and preserved with salt before being sealed in forensic evidence bags with a completed datasheet. Samples (2x2cm rib bone) were collected from i) farms, and ii) spot-checked skeletons at ORT airport (Fig 2), under an approved chain-of-custody procedure and sealed in forensic evidence bags with the details of each corresponding carcass. Evidence bags were hand-delivered to the SANBI laboratory and dry-stored at -20°C under lock-and-key until processed.

In total, the laboratory received 1,728 bone samples representing five sample sets: i) 800 farm samples in 2017, ii) 25 forensic exhibits from spot-checked port samples in 2017, iii) 800 farm samples in 2018, iv) 102 forensic exhibits from spot-checked port samples in 2018, and v) one sample collected from a skeleton confiscated at the airport in 2018 with the same tag number as an anomalous farm specimen (see S1 Table for summary of the number of samples collected and analysed). The delay in allocating the 2018 quota meant that the consignment spot-checks and exports for 2018 only occurred in early 2019, but were within the six-month CITES permit validity period.

A chain-of-custody protocol developed in consultation with the South African Police Service (SAPS), Environmental Management Inspectorate (EMI), and National Prosecuting Authority (NPA) for conducting forensic casework was followed to document the receipt, transfer, storage and opening of, as well as subsequent analysis from all exhibit bags to secure case traceability. Following serial photographic documentation, exhibit bags were opened, and bone samples duplicated under sterile conditions in a biosafety cabinet. A sub-sample was used for analysis, while the reference was stored in the SANBI Animal Biobank with accession numbers allocated for traceability (BON17/18/0001-0028). All associated documentation and metadata were stored under lock-and-key throughout.

DNA was extracted from the 1,728 bone samples using a PrepFiler™ BTA Forensic DNA extraction kit (Thermo Scientific, Massachusetts, USA), following the manufacturer’s guidelines on the KingFisher™ Duo Prime Purification System (Thermo Scientific, Massachusetts, USA), an automated magnetic bead-based DNA isolation instrument. Extracted DNA was quantified and its quality assessed by a NanoDrop 1000 Spectrophotometer (Thermo Scientific) before storage for later analyses at -20°C.

For species identification, a rapid, low-cost allelic discrimination real-time polymerase chain reaction (qPCR) assay was used to confirm lion and possible tiger in all samples, following the protocol described in Dalton et al. (2020) [26]. In summary, fixed nucleotide differences (Single Nucleotide Polymorphisms; SNPs) were identified in three mitochondrial genes (control region, 12S and 16S ribosomal RNA). DNA extracts (*n* = 1,728) were amplified using the validated SNP assay on an Applied Biosystems QuantStudio™ 12K Flex Real-time PCR system (Thermo Scientific). Positive control samples for lion (*P*. *leo*) and tiger (*P*. *tigris tigris*) were included in the genotyping with a negative control in each qPCR plate run to ensure no contamination or cross-amplification. Resulting species assignments were visualized through an SNP allelic discrimination plot.

If a non-lion or hybrid animal sample was suspected, the sample was re-analyzed, and species identification confirmed by i) qPCR, ii) mitochondrial DNA sequencing (DNA barcoding), and iii) microsatellite marker typing. First, an alternative analyst repeated the DNA extraction to qPCR steps above before amplifying the Cytochrome Oxidase I [27] and Cytochrome b [28] regions of the mitochondrial DNA using the Applied Biosystems 2720 Thermal Cycler. Total PCR amplification (25 μL) was conducted with: 1X Thermo Scientific DreamTaq Master Mix (2X), containing DreamTaq DNA polymerase and Green buffer, 4 mM MgCl_2_ and dNTPs; 10 pmol of each of the forward and reverse primers and 50–100 ng of genomic DNA template. Amplification conditions were: 5 min at 95°C (denaturation); 5 cycles of 30 sec at 95°C, 40 sec at 45°C, 1 min at 72°C; followed by 35 cycles of 30 sec at 95°C, 40 sec at 51°C, 1 min at 72°C; and a final extension for 10 min at 72°C. PCR products were separated by agarose electrophoresis (2% gel for 30 min at 100 volts in 1 x Tris-Acetate-EDTA Buffer) and cleaned using the ExoSAP protocol. Purified PCR products were cycle sequenced using the BigDye Terminator v3.1. Cycle Sequencing Kit (Life Technologies). The cycle sequencing products were purified with the BigDye XTerminator Purification Kit (Life Technologies) before sequencing on an ABI 3500 genetic analyzer (Life Technologies) in forward and reverse. Raw sequence data for each mtDNA gene were edited and aligned in GENEIOUS version R10 (https://www.geneious.com). Concatenated sequences were compared to known lion sequences available on Genbank (http://www.ncbi.nlm.gov/genbank), and queried in NCBI BLASTN using the MEGABLAST program, which is optimized for highly similar sequences [29], where query cover (%), unique identity (%), and top species accession matches were considered. To ensure that non-lion samples were not tiger x lion hybrids, these were duplicated and amplified using 23 microsatellite markers, along with 20 tiger and 20 lion reference samples for species assignment [19]. Total PCR amplification (12.5 μL) was conducted with: AmpliTaq® DNA polymerase (Roche Molecular Systems, Inc), forward and reverse primers (0.5 μM each) and 50 ng genomic DNA template. Amplification conditions were: 3 min at 94°C (denaturation); 10 cycles of 94°C for 30 s, 56°C for 30 s, 72°C for 30 s; 10 cycles of 94°C for 30 s, 51°C for 30 s, 72°C for 30 s; 20 cycles of 94°C for 30 s, 45°C for 30 s, 72°C for 30 s; and a final extension for 20 min at 72°C in a T100™ Thermal Cycler (Bio-Rad Laboratories, Inc. Hercules, CA, USA). Products were run against a Genescan™ 500 LIZ™ internal size standard on an Applied Biosystems™ 3500 Genetic Analyzer (Applied Biosystems, Inc., Foster City, CA, USA) and were genotyped using GeneMapper® 5 (Applied Biosystems, Inc., Foster City, CA, USA). A Bayesian clustering approach was implemented in STRUCTURE v2.3.4 [30–32] to assign individuals to species clusters. The model was run assuming admixture, correlated allele frequencies and without prior population information for ten replicates each with K = 2 (accounting for the two parental species), with a run-length of 700,000 Markov Chain Monte Carlo repetitions, following a burn-in period of 200,000 iterations. Species membership coefficient matrices (Q-matrices) of replicate runs for K = 2 was combined using CLUMPP v1.1.2 [33] with the FullSearch algorithm and G′ pairwise matrix similarity statistics before visualization using DISTRUCT v1.1 [34]. From K = 2, the average proportion of membership (qi) of the sampled populations to the inferred clusters confirmed species identity.

Once confirmed as lion, the 128 samples (*n*_2017_ = 25, *n*_2018_ = 103) from farm and port were further duplicated and re-amplified for pairwise-comparative sample matching using 18 validated microsatellite markers (S2 Table) to create unique DNA profiles. Total PCR amplification (12.5 μL) was conducted with: Ampliqon Taq DNA Polymerase Master Mix RED (Ampliqon, Odense, Denmark), forward and reverse primers (0.5 μM each) and 50 ng genomic DNA template. Amplification conditions were: 3 min at 94°C (denaturation); 10 cycles of 94°C for 30 s, 56°C for 30 s, 72°C for 30 s; 10 cycles of 94°C for 30 s, 51°C for 30 s, 72°C for 30 s; 20 cycles of 94°C for 30 s, 45°C for 30 s, 72°C for 30 s; and a final extension for 20 min at 72°C in a T100™ Thermal Cycler (Bio-Rad Laboratories, Inc. Hercules, CA, USA). Products were pooled and run against GenescanTM 500 LIZTM internal size standard on an Applied Biosystems™ 3500 Genetic Analyzer. Alleles were manually scored using GeneMapper 5 and markers assessed for their Polymorphic Information Content (PIC), probability of identity (PID) and exclusion (Pe1, one parent known and Pe2, both parents known), with null alleles calculated per locus in CERVUS [35]. Deviations from Hardy-Weinberg equilibrium (HWE) and linkage disequilibrium (LD) for each locus were determined using GENEPOP 3.4 [36]. The number of alleles (*A*_n_), effective alleles (*A*_e_), observed heterozygosity (*H*_o_) and expected heterozygosity (*H*_E_) per locus were determined in GenALEx [37]. Pairwise relatedness scores (Queller and Goodnight) [21] were generated across all samples by year, and significant matches between farm and port samples determined using generalized extreme studentized deviate (ESD) tests.

## Results

### Skull morphology

Species identification using comparative skull morphology indicated that all spot-checked samples were of lions (Fig 3A), except for one specimen in 2018 (confiscated on arrival at ORT airport; Fig 4), where the cranial sutures and mandibular curve resembled that of a tiger (Fig 3B), and was later confirmed as tiger by molecular methods.

### Skeleton weight

**Fig 5:**
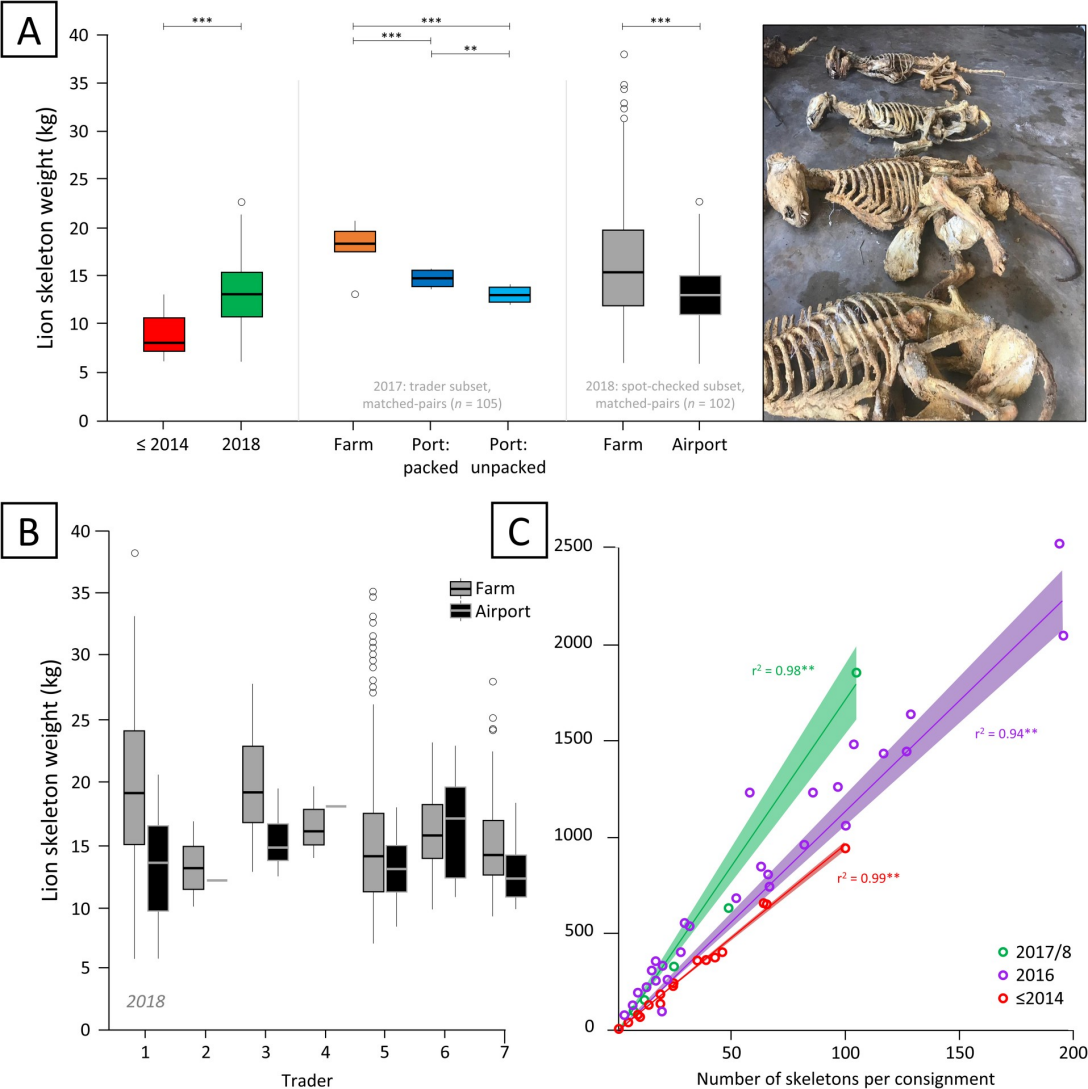
Lion skeleton weights by trader, location, consignment and year. (A) The difference in the mean weight (C.I. 95%) of skeletons recorded [9] at the airport before 2014 (red) compared to 2018 (green). The difference between mean farm (orange) and port skeleton weights packaged (dark blue) and unpackaged (light blue) for matched pairs in 2017. The difference in mean weight of matched-pair spot-checked skeletons weighed at source farms (grey) versus at O.R. Tambo airport (black) in 2018 (Table 1). (B) The differences in mean farm (grey) and airport (black) weights of spot-checked skeletons (12.5%) exported by seven traders in 2018. (C) The linear relationships between airport consignment weight and the number of skeletons in the consignment up to 2014 (red) and for 2016 (purple), as well as for 2017 and 2018 combined (green), where shading about the regression indicates the standard deviation. Note: unless specified otherwise, all port weights are for packaged skeletons.

Lion skeleton weights taken at farms (and recorded on CITES export permits) were significantly heavier than spot-check port weights for skeletons exported in 2018 (farms: ${\bar{x}}_{102}$ = 15.8 ± 5.5 kg; 6–38 kg; spot-checks: ${\bar{x}}_{102}$ = 13.5 ± 3.3 kg; 6–22.8 kg; *t*_*102*_ = 6.24, *P* < 0.001; Fig 5A; Table 1). Farm weighing took place prior ($\bar{x}$ = 9; 1–18 weeks) to export, thereafter skeletons were prepared (e.g., de-fleshed and cleaned), which resulted in a ± 12% weight reduction between farm (recorded on CITES permits) and port weights in 2018. Furthermore, packaging increased the weight of skeletons: in 2017, based on a subset of endorsed consignment weights for six consignments from one trader, the average packaged weight of skeletons was found to be significantly higher than the unpackaged weight (${\bar{x}}_{\mathrm{Difference}}$ = 1.10 ± 0.06 kg; *t*_5_ = 47.89*; P* < 0.001; Fig 5A). It should also be noted that there was found to be significant variation in skeleton weights between different lion bone traders based on data available for the year 2018 (farm weights: *F*_5_ = 14.87, *P* < 0.001; port weights: *F*_5_ = 2.40, *P* < 0.05; Fig 5B).

**Table 1:** Mean mass (kg) of captive-bred lion skeletons exported in 2017 and 2018 based on the weights recorded during the compliance inspections at source farms (all skeletons) and ORT airport.

	Farm			Spot-check^a^		
	$\bar{X}$ ± SD (kg)	*N*	Range (kg)	$\bar{X}$ ± SD (kg)	*N*	Range (kg)
*Lion*						
2017	15.2 ± 4.8	767^b^	6.2–35.0	-^c^	25	*-*
2018: all	16.6 ± 5.5	784^d^	6.0–38.0			
2018: spot-checked^e^^,^^f^	15.8 ± 5.5	102	6.0–38.0	13.5 ± 3.3	102	6.0–22.8
2017 & 2018 combined	15.9 ± 5.2	1551	6.0–38.0			
*Tiger*^*g*^	14	1	-	12.3	1	-

^a^ Equivalent to the final average weight (kg) of exported skeletons reflected on an air waybill (AWB).

^b^ Documentation misplaced for *n* = 33 lion skeletons.

^c^ Documentation not retained for *n* = 25 lion skeletons.

^d^ Documentation misplaced for *n* = 15 lion skeletons; since 799 skeletons were lions, 784 farm weights were analysed.

^e^ Mean decrease in skeleton weight between the farm and airport compliance inspections: 2.5 ± 4.0 kg (range: 0–17.9 kg).

^f^ Weights of 11 skeletons increased by an average of 0.5 ± 0.6 kg (0.05–2.1 kg); however, final export weights did not exceed 18.3kg.

^g^ DNA-based species assignment determined that the tiger identified at the source farm and ORT airport were the same individual.

Skeletons in more recent trade were found to have higher weights than those in preceding years: the mean weight of skeletons recorded at farms was significantly heavier in 2018 than 2017 (*t*_1524_ = 5.40*; P* < 0.001; Fig 5A; Table 1), and the mean weight of skeletons at port in 2018 was significantly heavier (*t*_102_ = -10.10*; P* < 0.001; Fig 5A) than those recorded up to 2014 [9]. In addition, consignment weights at port plotted against the number of skeletons per consignment showed a significant linear trend for the year groupings ≤*2014*, *2016*, and *2017/8*; linear regressions for these year groupings indicate an overall increase in consignment weight with a reduction in the number of individuals per consignment over time (Fig 5C).

All but one of the skeletons exported in 2018 were lions (${\bar{x}}_{784}$ = 16.6 ± 5.5 kg); the tiger skeleton (not exported) weighed 12.3 kg at port, falling within the variance exhibited by lions exported in that year (Table 1).

### Molecular analyses

**Fig 6:**
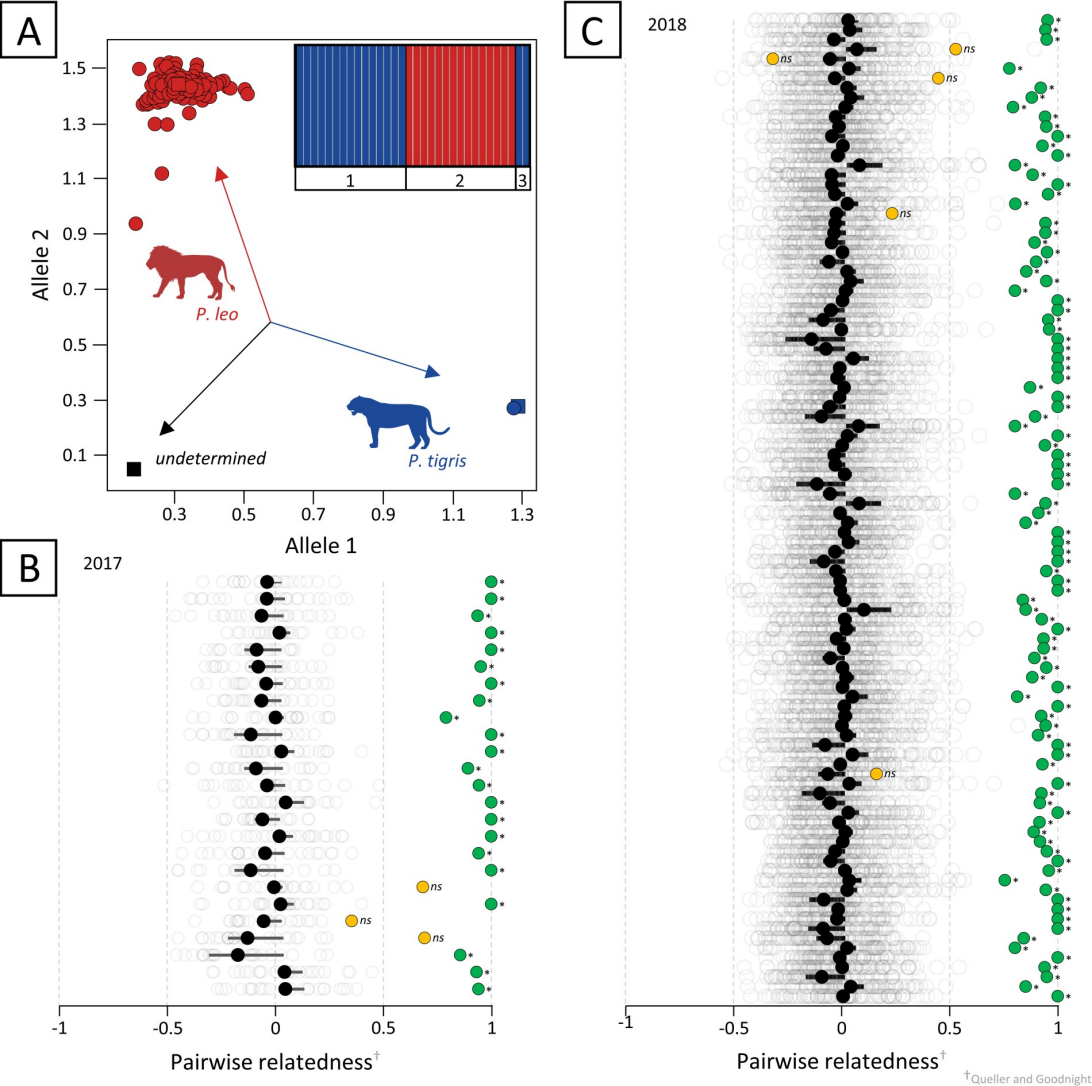
Species assignment and pairwise-comparative sample matching by relatedness scores between source farm and export consignments tags. Species is confirmed through (A) an allelic discrimination plot for 12S, where axes correspond to the relative fluorescence of labelled primers. Squares indicate positive (red, homozygous allele 1, lion; blue, homozygous allele 2, tiger) and negative controls (undetermined), where circles and colour represent sample assignments. Genetic differentiation between species based on STRUCTURE analysis (performed with K = 2) is also visualized using DISTRUCT (inset), indicating 1) tiger reference controls, 2) lion reference controls, and 3) the suspected tiger sample (in duplicate). Pairwise-comparative sample matching for the (B) 2017 and (C) 2018 quota indicate farm-port tag matches as significant (*P* < 0.05) deviations from the normal pairwise relatedness distribution (black whiskers) within comparisons (green) and those non-significant as unmatched and warranting further investigation (yellow).

The qPCR analysis confirmed 1,599 bone samples from farms in 2017 and 2018 as *P*. *leo*, with one sample being identified as *P*. *tigris* in 2018 (Table 2). Of the 127 spot-checked skeletons at ORT airport in 2017 and 2018, all were identified as *P*. *leo*, bar one identified as *P*. *tigris* (Fig 4E) confirmed by allelic discrimination for 12S (Fig 6A). Mitochondrial DNA sequencing for DNA barcoding was unsuccessful due to the co-amplification of nuclear DNA segments [38]. Microsatellite marker typing for species assignment identified a total of 149 alleles with 40 specific to tiger and 68 to lion. Posterior probabilities (Ln) using Bayesian admixture analysis indicated two distinct clusters with the unknown sample identified as pure *P*. *tigris* (Fig 6A).

**Table 2:** Number and proportion of identified sampling anomalies (species assignment and pairwise-comparative sample matching) in the DNA profiles by sampling location and year.

Year	All source farm	Airport spot-checked
2017	0/800 (0%)	3/25 (12%)^a^
2018	3/800 (0.3%)^b^	5/102 (4.9%)^c^

^a^ These samples did not match the skeletons with the same tag numbers sampled at source farms and were thus from different lions. DNA-based pairwise-comparative analyses revealed that these samples matched other lion skeletons sampled at the same farm from the 2017 quota.

^b^ One sample was identified as a tiger, and two samples had different tag numbers but were from the same individual lion skeleton.

^c^ One sample did not match the skeleton with the same source farm tag number and was thus from a different lion. DNA-based pairwise-comparative analyses revealed that this sample matched another lion skeleton sampled at the same farm from the 2018 quota. Four samples did not match the skeletons with the same tag numbers and were thus from different lions. DNA-based pairwise-comparative analyses revealed that these samples did not match other lion skeletons sampled at the same farm from the 2018 quota.

All 254 spot-checked samples (*n*_2017_ = 25; *n*_2018_ = 102; less the tiger sample and duplicated) were successfully amplified at 18 microsatellite loci for pairwise-comparative sample matching. No contamination or additional alleles were observed in any samples, with no loci showing evidence of null alleles, deviations from HWE or significant LD. The mean number of alleles (*A*_n_) was 7.5 (5–11), the overall observed heterozygosity (*H*_O_) was 0.619 and mean expected heterozygosity (*H*_E_) was 0.701, with the probability of identity across 18 markers at 1.38^−12^ and PIC values ranging from 0.189 to 0.797 (S3 Table). The genotype accumulation curve plateaued at five loci, indicating sufficient discriminatory power for individualization of the lion bone samples. Pairwise-comparative relatedness scores were calculated, and samples taken from farms were considered against their respective port sample. Of the 25 tags spot-checked on export in 2017, 22 (88%) were positive matches to the sampled farm carcasses, indicating that these originated from the same individual (Fig 6B; S4 Table). Where the DNA profiles did not match under the same tag numbers in 2017 (Table 2; S4 Table), it was found that three samples originated from a single captive breeding operation. All 142 samples submitted by this breeder were subsequently profiled, confirming positive matches within the batch, indicative of skeleton-pooling or mislabelled sampling prior to submission. Of the 102 tags, spot-checked on export in 2018, 97 (95%) were positive matches to their sampled farm carcasses, indicating that these originated from the same individual (Fig 6C; S5 Table). Five DNA profiles did not match under the same tag numbers (Table 2; S5 Table); one sample was unmatched, but further profiling identified a match from the same farm under a different tag number, indicative of skeleton-pooling or mislabelled sampling prior to submission; the remaining four samples showed no match across the remaining 796 farm samples, suggesting skeletons did not comprise a single individual and bones were ‘pooled’ under these tag numbers at the same farm.

## Discussion

To our knowledge, there are no peer-reviewed publications that evaluate commercial CITES compliance procedures of quota-based wildlife trade. The compliance procedure (Fig 1), including DNA-based testing, was introduced by the DEFF in 2017 to ensure stricter control of the CITES export permit process and, most importantly, to reduce the opportunities and incentives for illegal trade within the legal supply chain.

Molecular species identification confirmed all source and spot-checked skeleton exports as lion and successfully identified an attempt at laundering tiger bones (Table 2). Confirming species from a sample taken at the farm subsequently drove interception and confiscation of the skeleton at the airport, highlighting the value of this technique. The interception was possible due to a long interval between Steps 5 and 7 (Fig 1); leaving sufficient turnaround-time for the processing of DNA-based analyses before authorizing skeleton exports and is thus critical to ensuring compliance.

Molecular identification of individuals successfully highlighted ten anomalies in comparative sample matching of individual pairs with the same tag number between farms and ORT airport spot-checks. Of these anomalies, four were mismatches (i.e., the port sample matched a farm sample with a different tag number), four were port samples for which no pairs could be found among the farm samples, and two were farm samples ostensibly from the same lion (Table 2). It is essential to investigate further the circumstances surrounding irregularities, either at the farm or port spot-check, to determine their absolute cause and identify any weakness in the process, to ultimately improve sampling protocols and overall CITES compliance procedures–which may have broader applicability to quota systems for other species. We herewith identify two broad categories of circumstances that could potentially lead to these irregularities—bone mixing or pooling (Type I), and mislabelling or tag-swapping (Type II).

Type I has four potential sources of error: i) EMIs mixed samples between bags at Step 7, which is unlikely, as the chain of custody protocol only allowed for one skeleton to be processed at any time (V.L. Williams observed no deviations from this protocol), however, such ‘pooling’ of samples cannot be ruled out at Step 5 (Fig 1); ii) mixing error at the SANBI laboratory, which is unlikely given the forensic credibility of the laboratory and duplicated samples that were independently verified; iii) mixing of bones on source farms or trader warehouses before Step 7, meaning that a complete exported skeleton could comprise of multiple ‘pooled’ individuals; and iv) contamination of EMIs equipment when sampling bone samples for DNA analyses (Fig 2C), which is unlikely, as samples were tested for contamination. Thus, the most likely explanation for errors is mixing of bones on the farm before Step 5, which is supported by a trader who stated that on some farms skeletons are frequently stored in haphazard piles where inter-individual mixing occurs. Such practices were likely to have occurred in the absence of a prior directive from DEFF to suppliers of lion skeletons regarding the type of post-mortem treatment that each individual should receive. As some skeletons are stockpiled at farms for >1 year, mixing could have already occurred even after the issuance of a directive: further evidenced by the reduction in the number and therefore overall proportion of irregularities per 800 lion skeleton consignments between 2017 (12%) and 2018 (5%). While this may be ratified through DNA-based methods, these errors reinforce the need for clear and standardized information to be presented to all potential suppliers, allowing them sufficient opportunity to ensure product compliance.

Type II irregularities concern i) mislabelling of skeletons or ii) tag-swapping between Steps 5 and 7 –however, CITES tags are tamper-proof, making it unlikely they could have been physically switched between skeletons. Alternatively, the wrong tag may have been allocated on the source farm; however, this is also unlikely as EMIs were required to follow a strict protocol, processing only one skeleton at a time and maintaining an extensive photographic record of tagging, which includes relevant documentation with the same tag number. Such error, whether inadvertent or deliberate cannot be ruled out entirely.

Williams et al. (2015) [9] presented a morphometric method for the differentiation of tiger from lion skulls, which corroborated DNA-based evidence for the compliance procedure in 2018 (Fig 3). The tandem use of these methods in verifying species incurs minimal additional cost or delay and can conveniently be used *in situ* during compliance inspections, offering real-time results. This method requires a relatively low level of technical expertise and may be carried out by any EMI. However, such morphological techniques are not considered legally robust and must be used in conjunction with rigorous forensic procedures. Furthermore, an atypical feature on the mandibles of some specimens examined at ORT airport, that was not observed when compiling Williams et al. (2015) [9] for wild-origin specimens, suggested that there may be subtle variations in mandibular shape from captive-bred lions that, with further research, might make it possible to distinguish the source in future.

Although the monitoring of weight profiles can provide significant regulatory advantages when correctly applied, it may be prone to misinterpretation and should, therefore be considered in the full context of the procedural system (Fig 1). For instance, the use of CITES permit application weights (i.e., farm weights; Step 5) for post-hoc analysis (as in Pickover, 2019 [24]) should not be undertaken without paired weight data from the point of export (Step 7). This study [24] asserted that “*double the quantity of skeletons*” were exported in 2017. This assertion would have been based on farm weights alone–but as our research shows, however, i) there is a significant difference in weights taken at farms (recorded on the CITES permit) versus the final export weight taken at the port, where subsequent cleaning and desiccation account for such reductions, and ii) comparative sample matching of individuals from DNA profiles further suggests no more than the designated quota was exported. Nevertheless, when correctly applied, pairwise-weight profiles show promise as a complement to the compliance procedure. Future integration of this method should, however, be standardized to account for packaging significantly increasing the mean weight of skeletons (e.g., mean packaging weight per skeleton for one trader was 1.1 kg) and inaccurate equipment, where infrequent scale calibration and rounding errors will erroneously violate the >0.6 kg enforcement threshold in weight variance. Furthermore, the accuracy of the guide published in [9] for detecting consignment weight anomalies cannot be guaranteed for skeletons exported from 2016 onwards, given the year-on-year differences in mean skeleton weight (Fig 5C). Thus, regressions derived from skeleton weights in previous years are of limited use in determining the number of skeletons in a consignment. Steepening linear regression slopes reflect a significant increase in mean skeleton weight from before 2014 to 2018 (Fig 5A). Importantly, therefore, attempts to assess compliance using outdated regression equations will provide incorrect estimations and imply false compliance breaches. There is, however, a reliable predictive capability within years, where a known subset of consignments and skeleton weights could be used to assess compliance (number of individuals) within a quota. Nevertheless, a holistic, mixed-methods approach for the determination of compliance breaches is recommended. Year-on-year changes in weight profiles also provide insight into the wider lion farming industry. For example: i) the proportional contribution of trophy versus euthanized lions (notably the inclusion of skulls, which are typically not included with trophy hunted specimens); ii) reduced time between mortality and export in recent years; iii) the origin of skeletons; iv) demand-based preferences for the degree of processing (e.g., de-fleshed, packaged); and v) the influence of international political actions on trader behaviour [3].

From observing the implementation of the South African lion skeleton quota and compliance monitoring, we can draw several generalizable advisory points that may be applied to the establishment and management of other CITES Appendix II export quota systems. Although other quota systems will necessarily differ regarding specific details, we believe these six points may be essential to focus on when considering the implementation of similar systems:

1. *Information*–several potential errors in compliance with the lion quota could have been mitigated by the provision of more detailed information to the providers and traders of lion skeletons, especially regarding the pre-quota storage of skeletons, which led to mixing errors.
2. *Standardization–*some compliance errors would have been mitigated had equipment, procedure, monitoring task team and skeleton preparation been standardized, especially with regards to skeleton handling and packaging prior to export, as well as instrument type used between provinces and scale calibration.
3. *Cost*–DNA-based forensic analyses are relatively costly; it would have been preferable to have generated full profiles for every skeleton at Steps 5 and 7, and therefore eliminated spot-checking in favour of complete sampling. Such changes would have come at a substantial cost. However, this is strongly recommended given the gains in detection capability afforded for every skeleton.
4. *Data management*–system-wide analyses were complicated through missing records; this was unintentional as detailed weight analyses were only applied to the procedure post-hoc. However, complete record-keeping (and the retention thereof) over long periods are of clear benefit to the evaluation of compliance procedures.
5. *Timing*–the speed with which consigned skeletons progressed through the compliance procedure (from Steps 1 to 8) varied considerably, and in some cases of rapid turnaround (7–9 days) this could have led to non-compliant skeletons being exported; fortuitously it did not. Previous procedural steps must be fully completed before export is permitted to proceed.
6. *Product suitability*–a final complication is whether the exported product can be divided and reconstituted from multiple individuals or not. In the case of lion skeletons, this complicated the assignment of a sample taken from a single bone to an entire skeleton. Visual monitoring of skeletons by EMI/DEFF officials trained to recognize the appearance of multiple skeletons is therefore necessary in addition to DNA-based monitoring. It was found that extensive photographic records were invaluable in this regard.

## Conclusions

Monitoring of the legal lion bone trade in South Africa commenced in 2017 in response to the introduction of the lion bone quota. Accordingly, stricter control measures on the exports of lion skeletons were necessarily implemented by the DEFF to ensure compliance with decisions made by the CoP17. We reviewed this monitoring process for irregularities and non-compliance while assessing the relative value of current compliance monitoring techniques. There was only one overt attempt at illegal activity detected in 2018, which was at the farm before the tiger skeleton could be exported. Limited communication of relevant information to farmers and exporters, along with non-standardization of protocol, likely accounted for the remaining minor irregularities. The mixed-methods approach employed in South Africa (2017–2018) illustrated how sample anomalies could be detected and clarified timeously. Furthermore, evaluations of the protocol-related irregularities in the system enabled lessons to be learned for improving any future implementation and management of any CITES animal export quota, and securing a robust compliance procedure for legal lion bone exports.

This study demonstrates the applicability and importance of forensic molecular technologies in supporting compliance with export quota procedures. In the case of lions, these analyses reliably distinguished between lion and tiger species (corroborating the skull morphometric technique), and microsatellite markers were able to provide a robust and highly discriminatory test for individual identification and comparative sample matching per CITES tag between locations. However, the study also highlights the need for additional data verification procedures to be put in place. The use of weight to evaluate compliance requires full context of the procedural system and is not straightforward; and, our analyses indicated that heavier skeleton weights are not necessarily indicative of skeleton laundering.

We conclude by reiterating the need for research, development and verification of robust procedures for assessing compliance with CITES trade quotas. The legal trade in lion skeletons is a controversial topic under public scrutiny. Effective mechanisms for the prevention of illegal trade are essential not only for species survival but also to maintain trust in the authorities responsible for managing legal trade. We believe transparent and critical analysis of procedures designed to ensure compliance with legal trade requirements is essential in ensuring that emerging caveats are identified and used to improve future procedures. Although we focus on lion skeleton export, we hope that, should the need arise, insight can be drawn from this case to inform compliance procedures for regulated trade in other species.

## Supporting information

S1 Table Number of samples collected analysed per method. (XLSX)

S2 Table Microsatellite markers optimised and verified for sample matching derived from [19]. (XLSX)

S3 Table Summary statistics of 13 microsatellite loci. (XLSX)

S4 Table A heatmap showing relatedness values between all individual in the 2017 airport spot check database. (XLSX)

S5 Table A heatmap showing relatedness values between all individual in the 2018 airport spot check database. (XLSX)
